# Can the measurement of brachial artery flow-mediated dilation be applied to the acute exercise model?

**DOI:** 10.1186/1476-7120-5-45

**Published:** 2007-11-26

**Authors:** Jaume Padilla, Ryan A Harris, Janet P Wallace

**Affiliations:** 1 Clinical Exercise Physiology Laboratory, Department of Kinesiology, Indiana University, Bloomington, IN, USA; 2 Physiology Division, Department of Medicine, University of California, San Diego, La Jolla, CA, USA

## Abstract

The measurement of flow-mediated dilation using high-resolution ultrasound has been utilized extensively in interventional trials evaluating the salutary effect of drugs and lifestyle modifications (i.e. diet or exercise training) on endothelial function; however, until recently researchers have not used flow-mediated dilation to examine the role of a single bout of exercise on vascular function. Utilizing the acute exercise model can be advantageous as it allows for an efficient manipulation of exercise variables (i.e. mode, intensity, duration, etc.) and permits greater experimental control of confounding variables. Given that the application of flow-mediated dilation in the acute exercise paradigm is expanding, the purpose of this review is to discuss methodological and physiological factors pertinent to flow-mediated dilation in the context of acute exercise. Although the scientific rationale for evaluating endothelial function in response to acute exercise is sound, few concerns warrant attention when interpreting flow-mediated dilation data following acute exercise. The following questions will be addressed in the present review: Does the measurement of flow-mediated dilation influence subsequent serial measures of flow-mediated dilation? Do we need to account for diurnal variation? Is there an optimal time to measure post-exercise flow-mediated dilation? Is the post-exercise flow-mediated dilation reproducible? How is flow-mediated dilation interpreted considering the hemodynamic and sympathetic changes associated with acute exercise? Can the measurement of endothelial-independent dilation affect the exercise? Evidence exists to support the methodological appropriateness for employing flow-mediated dilation in the acute exercise model; however, further research is warranted to clarify its interpretation following acute exercise.

## Introduction

A marked increase in blood flow exerts a frictional force or shear stress on the endothelial surface causing the vessel to dilate [1]. On the basis of this physiological phenomenon, in 1992 Celermajer et al. [2] developed a non-invasive reactive hyperemia technique to assess endothelial function in the conduit artery. This technique utilizes a transient (5-min) suprasystolic forearm occlusion to generate a hyperemic-induced shear stress stimulus and subsequent vasodilation, which can be detected at the brachial artery via high-resolution ultrasound. The percent change from baseline diameter to post-occlusion diameter is calculated (flow-mediated dilation, FMD) and used as an index of endothelial function [2,3]. In 1995, Joannides and colleagues [4] were the first to examine the nitric oxide (NO) contribution to the FMD response in humans and found that NO blockade completely abolished FMD. At the moment, it is well accepted that FMD, when performed following the published guidelines [3], provides a functional bioassay for *in vivo *endothelium-derived NO bioavailability [5]. FMD is demonstrated to be reproducible [6-9], correlates with invasive measurements of endothelial function in the coronary arteries [10,11], and predicts future cardiovascular events [12,13]; other studies, however, have demonstrated that reduced FMD is a poor marker of the presence and severity of angiographically assessed coronary artery disease [14] and fails to show a prognostic value [15]. Albeit this conflicting evidence, FMD is still the preferred non-invasive outcome variable in intervention studies aimed at reducing atherosclerotic cardiovascular disease.

Although brachial artery FMD has been extensively utilized in intervention trials evaluating the salutary effect of drugs and lifestyle modifications (i.e. diet or exercise training) on vascular function [3], until recently researchers have not used FMD to examine the role of a single bout of exercise on NO-mediated endothelial function [16-24]. As advocated by Thompson et al. [25], it is possible that the effects of a bout of exercise (acute exercise) can predict the effects of chronic exercise (accumulation of single bouts of exercise over time), as is the case in several variables; blood pressure reduction being a prime example [25]. Utilizing the acute exercise model can be advantageous as it allows for an efficient manipulation of exercise variables (i.e. mode, intensity, duration, etc.) and permits greater experimental control of confounding variables. The acute exercise model can also be useful in investigating mechanisms of the exercise (acute or chronic) response. Given that the utilization of FMD in the acute exercise model is justified and emerging, we consider the discussion of methodological and physiological factors pertinent to FMD in the context of acute exercise timely. The purpose of the present review is to summarize the current literature on FMD in acute exercise and to discuss both the foundations and considerations applicable to the acute exercise model and FMD.

## Current literature on FMD in acute exercise

**Figure 1:**
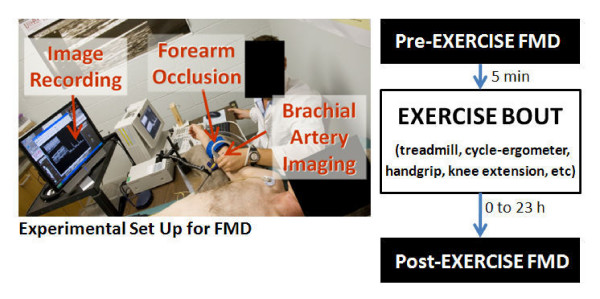
Representative experimental set up and design for evaluation of the FMD response to acute exercise.

Although the number of investigations reporting brachial artery FMD following exercise is scarce [16-24], the purpose for the study of post-exercise endothelial function and the diversity of the populations studied are not [see Additional file 1]. Oxidative stress [22], systemic and regional hemodynamics [18], postprandial endothelial function [19], endothelial response to acute exercise [16,23], inhaled particulate matter [24], and muscle ischemia [17] have been the focus of studies utilizing FMD in the acute exercise paradigm. Subjects have ranged from intercollegiate athletes [24] to apparently healthy adults [19] including pre- and post-menopausal women [18] and habitually sedentary men [20]; and to patients with claudication [17,21,22], renal disease [16], and who are overweight [23]. The breadth of journals publishing research articles utilizing the acute exercise model include cardiology [16,21,22], hypertension [18], exercise [20], thrombosis [17], obesity [23], physiology [19], and toxicology [24] foci. The only consistency found across all these studies is the use of treadmill walking/running as the mode of exercise. We consider treadmill exercise as a suitable mode due to its systemic effects and representation of the most common form of physical activity. Figure 1 illustrates a representative experimental set up and design for evaluation of the FMD response to acute exercise.

The acute exercise model has been utilized beyond the basic research studies listed in additional file 1. Investigators in the Framingham Study measured the post-exercise FMD in some of their epidemiological research on coronary heart disease [26]. Acute handgrip exercise has also been utilized during the occlusion phase to enhance ischemia and the subsequent hyperemic stimulus for FMD [27,28], or in substitution of occlusion [29]. The shear stress associated with one bout of exercise has also been observed by Tanaka et al. [30] and our group (data in review) in attempts to investigate the mechanism of exercise training-induced improvement of endothelial function. In addition, Gaenzer and colleagues [31] compared brachial artery vasodilation during cycle ergometry in smokers vs. nonsmokers. Thus, the use of the acute exercise model is even more diverse than the pre-to-post observation.

Questions arise from acute exercise studies in terms of the interaction of FMD and exercise. In most cases, only one post-exercise FMD measurement was performed [16-24], but on occasions FMD has been measured hourly following a treatment [32-34]. Do serial measurements of FMD influence subsequent measures of FMD? Do we need to account for diurnal variation? Is the post-exercise FMD reproducible?

Commonly, both measurements, FMD (endothelial-dependent dilation) and nitroglycerine-induced endothelial-independent dilation, are performed (within 10 min) [3] before and after a long-term treatment in order to determine whether changes in FMD are truly reflective of changes in endothelial function. When measurements of the endothelial dependent/independent dilation are applied to the acute exercise model, the following question arises: What is the influence of the nitroglycerine on the subsequent bout of exercise? If the exercise is delayed to account for washout of nitroglycerine, can the subsequent FMDs be compared to the baseline? Only three investigators [18,20,22] measured endothelial independent dilation with the use of nitroglycerine; one on a separate day [22], and the other two following both the pre- and post-exercise FMD measurements [18,20]. Neither investigator who administered nitroglycerine before exercise [18,20] reported the time between the endothelial-independent dilation measurement and exercise or addressed this issue. How can the separation of one day between the measurements of dependent and independent pathways be interpreted?

Exercise evokes hemodynamic and sympathetic changes which, in turn, may influence FMD. How is FMD interpreted considering these physiological changes associated with acute exercise? The timing of the measurement of the post-exercise FMD ranged from immediately post [16] to 23 hours post-exercise [20]. Is there an optimal time to measure post-exercise FMD?

A diversity of interest in the acute exercise paradigm is evident from the current literature. For this reason, we consider the discussion of the foundations and considerations of applying FMD to the acute exercise model timely.

## Foundations for FMD in the acute exercise model

The foundations to apply FMD to the acute exercise model have been established. First, similar to diet [34,35] and pharmacological research [36,37], the study of acute effects of exercise requires serial measurements to be taken in the same day but at succeeding times. Evidence suggests that serial FMD measurements do not affect subsequent FMD outcomes [38]. Second, diurnal variation has not posed a methodological problem in studies of less than two hours between FMD measurements [38]. Third, reproducibility has been well documented for FMD measurements [6-9] and recently for the acute exercise model [33].

### Serial Measurements of FMD

To investigate the influence of serial measures of FMD, brachial artery FMD was measured every 30 min (T_30_), every 60 min (T_60_), and every 120 min (T_120_) on three separate, randomized treatment days for a two hour period, beginning at 8:00 am; totaling 5, 3, and 2 measurements respectively. Subjects were 16 healthy young adults (9 men, 7 women), age 22.5 ± 0.8 yrs. The first (8:00 am) and last (10:00 am) FMD of each treatment time period were analyzed with ANOVA and intra-class correlations (ICC). No significant differences (p > 0.05) in FMD were found between the first (F) and last (L) measurements, within (p = 0.838) and between (p = 0.819) treatments. The ICC for all treatments combined was 0.7113. The ICC_F-L _for T_30_, T_60_, and T_120 _were 0.6068, 0.7415, and 0.7956 respectively. Thus, it appears that repetitive reactive hyperemia does not affect the FMD outcome [38].

### Diurnal Variation of FMD

In order to ensure that the FMD measurements are not influenced by diurnal variation, two different approaches from a study-design standpoint can be undertaken. First, the use of a non-intervention control day may be employed. This approach has been extensively used to investigate the acute effects of interventions on other physiological variables (e.g. blood pressure) that also exhibit diurnal variations [39-41]. For FMD, the use of a control day may not be suitable due to the day-to-day FMD variability [8]. Second, it is the evaluation of the FMD response to exercise during a time of day that shows evidence of non-variable FMD. The examination of FMD was performed in 20 healthy young adults (age = 23.1 ± 0.73 yrs) every 30 minutes from 8 am to 10 am [38]. Subjects exhibited a stable FMD response throughout the two hour morning period (p = 0.348). These results suggested that there is minimal variation during the two-hour morning period. A prolonged period of time, however, needs to be examined to further describe the diurnal rhythm of FMD.

### Reproducibility of FMD

The reproducibility of FMD following exercise was measured in nine middle-aged (56.4 ± 7.0 yrs), sedentary overweight men who performed two bouts of treadmill walking at 50% of VO_2_peak for 45 min (separate days) [33]. FMD was measured pre- and post-exercise (hourly for three hours). The two trials were within the acceptable 2–3% variation reported in the guidelines for brachial artery FMD measurement [3]. In addition, the intra-class correlation coefficient (ICC) for the difference in post-exercise FMD from baseline between the two trials was 0.595 (p = 0.028). Thus, the reproducibility for FMD following moderate intensity exercise is within the recommendations for resting brachial artery FMD measurements.

Thus, evidence in serial measurements, diurnal variation, and reproducibility support the methodological appropriateness for employing FMD in the acute exercise model. Beyond these methodological concerns; however, other physiological considerations now emerge.

## Considerations for FMD in the acute exercise model

Physical exercise is a physiological stimulus evoking a variety of transient biological alterations (i.e. blood flow, vascular tone, sympathetic activity, etc.) that should be, at the minimum, considered when interpreting the FMD response to exercise. Factors that are related to the FMD as the outcome measure as well as aspects referring to the mechanism of the FMD response will be discussed.

First, exercise-induced alteration of arterial diameter should be considered. The brachial artery is exposed to an increase in blood flow albeit work is performed by the lower extremities [30]. This augmented blood flow, through local regulatory mechanisms, causes vasodilation; which can persist for at least 1 h after cessation of exercise [23,42]; depending on the exercise intensity [23]. Variability in the magnitude of the arterial diameter has been recognized to limit interpopulation comparison studies [43] as large pre-occlusion diameters are associated with low FMDs and vice versa due to the mathematical bias of the FMD equation[43]:

[(peak diameter - baseline diameter)/baseline diameter] × 100

**Figure 2:**
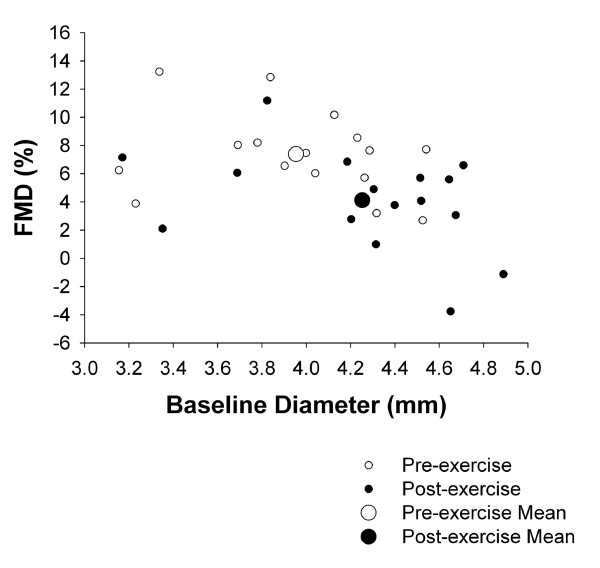
The influence of exercise on baseline diameters and subsequent FMD. Exercise (45 min @ 75% VO_2_max) was performed by 12 middle aged overweight men on a treadmill. FMD measurements were obtained before and immediately (<10 min) post-exercise.

Unfortunately, an analogous situation occurs when pre- to post-acute exercise FMD comparisons are being performed. In this context, a post-exercise dilated artery may be associated with a relatively low FMD; hence, complicating appropriate interpretation of the results. This concern may be amplified when FMD measurements are performed immediately following cessation of exercise where vasodilation may be maximal. Unpublished data in our laboratory shows that brachial artery FMD is decreased immediately post-treadmill exercise (45 min) in an intensity dependent manner. Although it is possible that this response is mediated, at least partially, by exercise-induced oxidative stress [44], it is likely that the vasodilation associated with exercise contributes to the attenuation in FMD. Figure 2 illustrates brachial artery vasodilation immediately post-exercise (right shift of data points) and the associated reduction in FMD (down shift of data points).

Second, the influence of exercise-induced shear stress deserves attention. Recently, our group found brachial artery shear stress to remain elevated for two hours following walking exercise (data in review). Superimposing the exercise- and reactive hyperemia-induced shear stress may modify the overall stimulus for FMD. Although Pyke and colleagues [45-47] have developed attractive methods to control for the variability in reactive hyperemic shear stress, the impact of an altered pre-hyperemic shear on the FMD measurement has not been clarified. Original statistical solutions are now being proposed [48] but further endeavor is warranted.

Last, the impact of exercise-induced sympathetic activity modulation on FMD should be addressed as it may alter the mechanism of the FMD response. The sympathetic nervous system is critical in maintaining cardiovascular homeostasis in humans [49]. Researchers have used a number of acute stimulations (i.e. cold pressor test, mental stress, low body suction) to investigate the impact of elevated sympathetic activity on the vasculature and they have observed a transient reduction in FMD following the stress [50,51]; although findings appear to be dependent on the nature of the stimulus [52]. In a recent study, Thijssen et al. [42] utilized leg exercise to locally reduce sympathetic responsiveness (functional sympatholysis) and found that older men were able to restore their blunted FMD in the femoral artery. Given that sympathetic nerve activity is decreased for several hours following a bout of exercise [53,54], it is possible that improvements observed in FMD following acute exercise can be partially explained by attenuation in sympathetic outflow, and may not necessarily reflect enhanced NO bioavailability. Interestingly, Harvey et al. [18] reported a significant increase in brachial artery FMD following acute exercise in post-menopausal women, whereas no change was observed in young pre-menopausal women. Providing that sympathetic nerve activity increases with advancing age [55], it is not unreasonable to speculate that improvement of FMD found in the older women [18] could be attributed to reduction in sympathetic outflow following exercise. In other words, in physiological states where alteration of sympathetic activity likely exists (i.e. post-exercise), caution should be taken when making the link between FMD and NO bioavailability. In this post-exercise context, it is possible that FMD represents a combined reflection of NO bioavailability and sympathetic modulation.

Collectively, the unequal pre- to post-acute exercise hemodynamic and sympathetic states may complicate FMD comparisons and interpretations associated with exercise. It is also possible that the exercise-associated changes in FMD may not well reflect actual NO-mediated endothelial function changes.

Furthermore, concerns regarding proper interpretation of acute exercise and FMD data and accurate comparison of findings among studies may simply result from substantial discrepancies in FMD protocols utilized across labs. In an effort to standardize the FMD measurement among researchers, guidelines for the use of FMD were published in 2002 [3]; however, it appears that further work is needed to ensure consistency in methodology. In 2005, Pyke and Tschakovsky [46] provided thorough reasoning on the serious consequences of varying the FMD protocol on interpretation of the data. They reviewed the implications of several FMD technique modifications, including placement of cuff occlusion, duration of cuff occlusion, addition of ischemic handgrip exercise, and location of ultrasound prove (brachial versus radial artery). In addition, inconsistency exits in the literature regarding the timing of post-occlusion diameter measurements. The fix measurement at the 60-second mark has been traditionally used [32,34]; however, some data suggest that the time-to-peak response may be longer in disease populations [56]. This problem is currently solved with the utilization of wall-tracking software capable of monitoring the entire time course response.

## Future research and conclusion

Further research is warranted to validate the use of FMD with acute exercise. In particular, future investigations should 1) examine the FMD diurnal rhythm and determine longer time periods of stable FMD; 2) identify the optimal time to measure post-exercise FMD; 3) compare the effect of different modalities of acute exercise on FMD to determine whether exercise produces a localized or systemic effect; 4) determine whether the measurement of endothelial-independent dilation (use of nitroglycerine) affects the exercise bout; 5) resolve the influence of exercise-induced vasodilation (change in baseline diameters) and/or augmented shear stress on the FMD outcome; and 6) determine the impact of exercise-induced sympathetic activity modulation on the mechanism of the FMD response. The later questions could be approached by incorporating endothelial NO synthase inhibitors (i.e. L-NMMA) into the FMD measurements before and after exercise. Is the abolishment of FMD induced by L-NMMA at post-exercise the same as that found pre-exercise? Inhibition of endothelial NO synthase would provide with evidence of the presence or absence of non-NO factors potentially influencing the FMD response to exercise.

In addition, studies are needed to determine whether changes in FMD induced by acute exercise are accompanied with changes in other accepted markers of endothelial function such as circulating adhesion molecules (ICAM-1, VCAM-1, E-selectin) and Von Willebrand factor [57]. Surprisingly, only one out of all published studies [21] evaluating the effect of acute exercise on FMD has reported other markers of endothelial function. In this study, Silvestro et al. [21] found soluble VCAM-1 to mildly accompany changes in FMD induced by acute exercise in combination with a pharmacological treatment; whereas soluble ICAM-1 did not follow the FMD pattern. Correlation analysis between delta change in FMD and soluble VCAM-1 was not performed. Although it can only be speculated, we are concerned that, if in fact more studies examining multiple endothelial markers do exist, the lack of association between FMD and these markers may be the cause of this gap of information in the literature. Unfortunately, it is indisputable that studies failing to find an association or lacking statistical significance are of less interest.

In conclusion, the scientific rationale for evaluating endothelial function in response to acute exercise is sound. Evidence exists supporting the methodological appropriateness for employing FMD as an outcome measure in the acute exercise model. Few concerns, however, warrant attention when interpreting FMD data following acute exercise.

## Authors' contributions

JP and JPW significantly contributed to manuscript writing and RAH significantly revised the manuscript for important intellectual content. All authors read and approved the final manuscript.

## Supplementary Material

Additional File 1 A summary of brachial artery FMD research articles utilizing the acute exercise model. This is a table summarizing all the exercise studies using FMD Click here for file

## References

[B1] Pohl U, Holtz J, Busse R, Bassenge E (1986). Crucial role of endothelium in the vasodilator response to increased flow in vivo. Hypertension.

[B2] Celermajer DS, Sorensen KE, Gooch VM (1992). Non-invasive detection of endothelial dysfunction in children and adults at risk of atherosclerosis. Lancet.

[B3] Corretti MC, Anderson TJ, Benjamin EJ, Celermajer D, Charbonneau F, Creager MA, Deanfield J, Drexler H, Gehard-Herman M, Herrington D, Vallance P, Vita J, Vogel R (2002). Guidelines for the Ultrasound Assessment of Endothelial-Dependent Flow-Mediated Vasodilation of the Brachial Artery. Journal of American College of Cardiology.

[B4] Joannides R, Haefeli WE, Linder L, Richard V, Bakkali E, Thuillez C, Luscher TF (1995). Nitric oxide is responsible for flow-mediated dilation of human peripheral conduit arteries in vivo. Circulation.

[B5] Green D (2005). Point: Flow-mediated dilation does reflect nitric oxide-mediated endothelial function. Journal of Applied Physiology.

[B6] Sorensen KE, Celermajer D, Spiegelhalter D (1995). Non-invasive measurement of human endothelium dependent arterial responses: accuracy and reprocucibility. Bristish Heart Journal.

[B7] Welsch MA, Allen JD, Geaghan JP (2002). Stability and reproducibility of brachial artery flow-mediated dilation. Medicine and Science of Sport and Exercise.

[B8] West SG, Wagner P, Schoemer SL, Hecker KD, Hurston KL, Likos Krick A, Boseska L, Ulbrecht J, Hinderliter AL (2004). Biological correlates of day-to-day variation in flow-mediated dilation in individuals with Type 2 diabetes: a study of test-retest reliability. Diabetologia.

[B9] Donald AE, Charakida M, Cole TJ, Friberg P, Chowienczyk PJ, Millasseau SC, Deanfield JE, Halcox JP (2006). Non-invasive assessment of endothelial function: which technique?. Journal of the American College of Cardiology.

[B10] Anderson TJ, Uehata A, Gerhard MD (1995). Close relationship of endothelial function in the human coronary and peripheral circulations. Journal of American College of Cardiology.

[B11] Takase B, Uehata A, Akima T, Nagai T, Nishioka T, Hamabe A, Satomura K, Ohsuzu F, Kurita A (1998). Endothelium-dependent flow-mediated vasodilation in coronary and brachial arteries in suspected coronary artery disease. American Journal of Cardiology.

[B12] Brevetti G, Silvestro A, Schiano V, Chiariello M (2003). Endothelial dysfunction and cardiovascular risk prediction in peripheral arterial disease. Circulation.

[B13] Widlansky ME, Gocke N, Keaney JF, Vita JA (2003). The clinical implications of endothelial dysfunction. Journal of American College of Cardiology.

[B14] Palinkas A, Toth E, Amyot R, Rigo F, Venneri L, Picano E (2002). The value of ECG and echocardiography during stress testing for identifying systemic endothelial dysfunction and epicardial artery stenosis. European Heart Journal.

[B15] Venneri L, Poggianti E, Jambrik Z, Varga A, Palinkas A, Picano E (2007). The elusive prognostic value of systemic endothelial function in patients with chest pain syndrome. International Journal of Cardiology.

[B16] Cosio-Lima LM, Thompson PD, Reynolds KL, Headley SA, Winter CR, Manos T, Lagasse MA, Todorovich JR, Germain M (2006). The acute effect of aerobic exercise on brachial artery endothelial function in renal transplant recipients. Preventive Cardiology.

[B17] Gresele P, Migliacci R, Procacci A, De Monte P, Bonizzoni E (2007). Prevention by NCX 4016, a nitric oxide-donating aspirin, but not by aspirin, of the acute endothelial dysfunction induced by exercise in patients with intermittent claudication. Thrombosis and Haemostasis.

[B18] Harvey PJ, Beverley LM, Kubo T, Picton PE, Su WS, Catherine FN, Floras JS (2005). Hemodynamic after-effects of acute dynamic exercise in sedentary normotensive postmenopausal women. Journal of Hypertension.

[B19] Padilla J, Harris RA, Fly AD, Rink LD, Wallace JP (2006). The effect of acute exercise on endothelial function following a high-fat meal. European Journal of Applied Physiology.

[B20] Pullin CH, Bellamy MF, Bailey D, Ashton M, Davies B, Williams S, Goodfellow J, Wilson JF, Lewis MJ (2004). Time course of changes in endothelial function following exercise in habitually sedentary men. Journal of Exercise Physiology.

[B21] Silvestro A, Schiano V, Bucur R, Brevetti G, Scopacasa F, Chiariello M (2006). Effect of propionylcarnitine on changes in endothelial function and plasma levels of adhesion molecules induced by acute exercise in patients with intermittent claudication. Angiology.

[B22] Silvestro A, Scopacasa F, Oliva G, Cristofaro T, Iuliano L, Brevetti G (2002). Vitamin C prevents endothelial dysfunction induced by acute exercise in patients with intermittent claudication. Atherosclerosis.

[B23] Harris RA, Padilla J, Hanlon KP, Rink LD, Wallace JP (2007). The Interaction of IL-6 and TNF-a on the FMD response to acute exercise in overweight active and inactive men. Obesity Research.

[B24] Rundell KW, Hoffman JR, Caviston R, Bulbulian R, Hollenbach AM (2007). Inhalation of ultrafine and fine particulate matter disrupts systemic vascular function. Inhalation Toxicology.

[B25] Thompson PD, Crouse SF, Goodpaster B, Kelley D, Moyna NM, Pescatello L (2001). The acute versus chronic response to exercise. Medicine and Science in Sports and Exercise.

[B26] Benjamin EJ, Larson MG, Keyes MJ, Mitchell GF, Vasan RS, Keaney JF, Lehman BT, Fan S, Osypiuk E (2004). Clinical correlates and heritability of flow-mediated dilation in the community: the Framingham Heart Study. Circulation.

[B27] Agewall S, Hulthe J, Fagerberg B, Gottfridsson B, Wikstrand J (2002). Post-occlusion brachial artery vasodilatation after ischaemic handgrip exercise is nitric oxide mediated. Clinical Physiology and functional imaging.

[B28] Wendelhag I, Fagerberg B, Wikstrand J (1999). Adding ischaemic hand exercise during occlusion of the brachial artery increases the flow-mediated vasodilation in ultrasound studies of endothelial function. Clinical Physiology.

[B29] Padilla J, Harris RA, Fly DA, Rink LD, Wallace JP (2006). A comparison between active- and reactive-hyperaemia-induced brachial artery vasodilation. Clinical Science.

[B30] Tanaka H, Shimizu S, Ohmori F, Muraoka Y, Kumagai M, Yoshizawa M, Kagaya A (2006). Increases in blood flow and shear stress to nonworking limbs during incremental exercise. Medicine and Science of Sport and Exercise.

[B31] Gaenzer H, Neumayr G, Marschang P, Sturm W, kirchmair R, Patsch JR (2001). Flow-mediated vasodilation of the femoral and brachial artery induced by exercise in healthy nonsmoking and smoking men. Journal of American College of Cardiology.

[B32] Bae J, Schwemmer M, Lee I, Lee H, Park K, Kim K, Bassenge E (2003). Postprandial hypertriglyceridemia-induced endothelial dysfunction in healthy subjects is independent of lipid oxidation. International Journal of Cardiology.

[B33] Harris RA, Padilla J, Hanlon KP, Rink LD, Wallace JP (2007). Reproducibility of the flow-mediated dilation response to acute exercise in overweight men. Ultrasound in Medicine and Biology.

[B34] Vogel RA, Corretti MC, Plotnick GD (1997). Effect of a single high-fat meal on endothelial function in healthy subjects. American Journal of Cardiology.

[B35] Tsai W, Li Y, LIn C, Chao T, Chen J (2004). Effects of oxidative stress on endothelial function after a high-fat meal. Clinical Science.

[B36] Aznaouridis KA, Stamatelopoulos KS, Karatzis EN, Protogerou AD, Papamichael CM, Lekakis JP (2007). Acute effects of renin-angiotensin system blockade on arterial function in hypertensive patients. Journal of Human Hypertension.

[B37] Eskurza I, Myerburgh LA, Kahn ZD, Seals DR (2005). Tetrahydrobiopterin augments endothelium-dependent dilatation in sedentary but not in habitually exercising older adults. Journal of Physiology.

[B38] Harris RA, Padilla J, Rink LD, Wallace JP (2006). Variability of flow-mediated dilation measurements with repetitive reactive hyperemia. Vascular Medicine.

[B39] Padilla J, Wallace JP, Park S (2005). Accumulation of physical activity reduces blood pressure in pre- and hypertension. Medicine and Science of Sport and Exercise.

[B40] Park S, Rink LD, Wallace JP (2006). Accumulation of physical activity leads to a greater blood pressure reduction than a single continuous session, in prehypertension. Journal of Hypertension.

[B41] Wallace JP, Bogle PG, King BA, Krasnoff JB, Jastremski CA (1999). The magnitude and duration of ambulatory blood pressure reduction following acute exercise. Journal of Human Hypertension.

[B42] Thijssen DH, de Groot P, Kooijman M, Smits P, Hopman MT (2006). Sympathetic nervous system contributes to the age-related impairment of flow-mediated dilation of the superficial femoral artery. American Journal of Physiology.

[B43] Uehata A, Lieberman EH, Gerhard MD, Anderson TJ, Ganz P, Polak JF, Creager MA, Yeung AC (1997). Noninvasive assessment of endothelium-dependent flow-mediated dilation of the brachial artery. Vascular Medicine.

[B44] Suvorava T, Kojda G (2007). Prevention of transient endothelial dysfunction in acute exercise: A friendly fire?. Thrombosis and Haemostasis.

[B45] Pyke KE, Dwyer EM, Tschakovsky ME (2004). Impact of controlling shear rate on flow-mediated dilation responses in the brachial artery of humans. Journal of Applied Physiology.

[B46] Pyke KE, Tschakovsky ME (2005). The relationship between shear stress and flow-mediated dilatation: implications for the assessment of endothelial function. Journal of Physiology.

[B47] Pyke KE, Tschakovsky ME (2007). Peak vs. total reactive hyperemia: which determines the magnitude of flow-mediated dilation?. Journal of Applied Physiology.

[B48] Harris RA, Padilla J (2007). Proper "normalization" of flow-mediated dilation for shear. Journal of Applied Physiology.

[B49] Seals DR, Dinenno FA (2004). Collateral damage: cardiovascular consequences of chronic sympathetic activation with human aging. American Journal of Physiology - Heart and Circulatory Physiology.

[B50] Hijmering ML, Stroes ES, Olijhoek J, Hutten BA, Blankestijn PJ, Rabelink TJ (2002). Sympathetic activation markedly reduces endothelium-dependent, flow-mediated vasodilation. Journal of American College of Cardiology.

[B51] Lind L, Johansson K, Hall J (2002). The effects of mental stress and the cold pressure test on flow-mediated vasodilation. Blood Pressure.

[B52] Dyson KS, Shoemaker JK, Hughson RL (2006). Effect of acute sympathetic nervous activitation on flow-mediated dilation of brachial artery. American Journal of Physiology - Heart and Circulatory Physiology.

[B53] Floras JS, Sinkey CA, Aylward PE, Seals DR, Thoren PN, Mark AL (1989). Postexercise hypotension and sympathoinhibition in borderline hypertensie men. Hypertension.

[B54] Pober DM, Braun B, Freedson PS (2004). Effects of a single bout of exercise on resting heart rate variability. Medicine and Science of Sport and Exercise.

[B55] Ng AV, Callister R, Johnson DG, Seals DR (1993). Age and gender influence muscle sympathetic nerve activity at rest in healthy humans. Hypertension.

[B56] Palinkas A, Toth E, Venneri L, Rigo F, Csanady M, Picano E (2002). Temporal heterogeneity of endothelium-dependent and -independent dilatation of brachial artery in patients with coronary artery disease. International Journal of Cardiovascular Imaging.

[B57] Constans J, Conri C (2006). Circulating markers of endothelial function in cardiovascular disease. Clinica Chimica Acta.

