# Supplementary material for: Can the measurement of brachial artery flow-mediated dilation be applied to the acute exercise model?
Source: Cardiovasc Ultrasound. 2007 Nov 26;5:45. doi: 10.1186/1476-7120-5-45 (PMC2211283; doi:10.1186/1476-7120-5-45)
Supplement: Additional File 1 — A summary of brachial artery FMD research articles utilizing the acute exercise model. This is a table summarizing all the exercise studies using FMD [file 1476-7120-5-45-S1.doc]

| **A summary of brachial artery FMD research articles utilizing the acute exercise model.** | | | | | | | |
| --- | --- | --- | --- | --- | --- | --- | --- |
| **Author** | **Purpose** | **Subjects** | **Exercise** | **Measurement Intervals** | **Measurement of Independent Pathway** | **FMD Results** | **Comments** |
| Silvestro et al., 2002 [22] | To investigate oxidative stress associated with exercise & endothelial function in claudication patients | - 31 claudication patients divided into two subgroups   - Maximal exercise   - Submaximal exercise - 10 age & sex matched controls | - Treadmill GXT - Maximal: to intolerance of claudication pain - Submaximal: to onset of claudication | - Baseline - 5 min Post | On a separate day | - Controls @ maximal exercise 12.9±1.1%→14.1±1.4% - Patients @ maximal exercise 8.5±0.9%→3.7±0.8%* - Patients @ submaximal exercise 7.3±1.1%→9.0± 1.0% | Used Vitamin C to counteract oxidative stress associated with exercise. |
| Pullin et al, 2004 [20] | To determine the time course of endothelial function following exercise in sedentary men | - 11 sedentary men | - Bruce Protocol for Treadmill (maximal testing) - Daily for five days - No exercise for 6 days | - Baseline - 23 hours post - Daily for six days during training - Daily for four days during detraining | Following each FMD measurement | - FMD increased steadily each day; significant at day 3 and maximal at day 6. - FMD returned to baseline on day 9 |  |
| Harvey et al., 2005 [18] | To determine the effects of exercise on systemic and regional hemodynamics in sedentary normotensive postmenopausal women | - 13 postmenopausal women - 14 premenopausal women | - Treadmill - 45 min @ 60% VO_2_max | - Baseline - 45-90 min postexercise | After both pre- and post-exercise FMD measurements | - Postmenopausal 5.3±1.3%→9.9±1.4%* - Premenopausal 12.1±1.5%→14.4±1.2% |  |

| Table 1 continued | | | | | | | |
| --- | --- | --- | --- | --- | --- | --- | --- |
| **Author** | **Purpose** | **Subjects** | **Exercise** | **Measurement Intervals** | **Measurement of Independent Pathway** | **FMD Results** | **Comments** |
| Padilla et al., 2006 [19] | To test the ability of exercise to counteract the postprandial endothelial dysfunction associated with a high-fat meal. | - 8 Apparently Healthy | - Treadmill - 45 min @ 60% VO_2_max | - Baseline – (2.5 hours before exercise) - 1 hr Post | None | - 5.61±1.5%→8.72±0.94%* | Exercise followed a high-fat meal; exercise was not performed by itself. |
| Silvestro et al., 2006 [21] | To evaluate the efficacy of intravenously administered propionylcarnitine (PLC) in preventing exercise-induced endothelial dysfunction in patients with claudication | - 36 claudication pt - 18 received PLC - 18 received Placebo | - Treadmill GXT - Maximal: to intolerance of claudication pain | - Baseline - 5 min Post | None | - PLC group 7.1±0.7%→6.0±0.6% - Placebo group 6.5±0.4%→4.4±0.5%* | Only study to measure sVCAM-1 and sICAM-1 |
| Cosio-Lima et al., 2006 [16] | To determine if FMD improved following acute exercise in renal disease patients | - 11 renal transplant patients - 11 controls | - Treadmill - 30 min - RPE 13 | - Baseline - Immediate Post | None | - Control 3.4±5.9%→22.3±13.2%* - Renal Patients 0.9±+3.1%→3.3±4.4%*^#^ | Artery diameters were measured 1,3 and 15 min post occlusion; the 1 min was reported as significant |

| Table 1 continued | | | | | | | |
| --- | --- | --- | --- | --- | --- | --- | --- |
| **Author** | **Purpose** | **Subjects** | **Exercise** | **Measurement Intervals** | **Measurement of Independent Pathway** | **FMD Results** | **Comments** |
| Rundell et al., 2007 [24] | Investigated the effects of inhaled particulate matter on conduit artery function | - 16 intercollegiate athletes - men | - 30 min running @ 85-90% of maximal heart rate on   - Inner campus jogging trail   and   - - Soccer field next to highway | - Baseline - 20-30 min post | None | - 6.6±4.04→4.9±4.22% for jogging trail - 6.8±3.58→0.3±2.74%* for field next to highway |  |
| Gresele et al, 2007 [17] | Compare treatment with aspirin vs. Nitroaspirin in claudication | - 44 patients with intermittent claudication | - Treadmill walking @ 3 km/h, 10% - Walking distance limited by claudication | - 10 min post | None | - -0.5 % change in arterial diameter in Aspirin group - +2.0% change in arterial diameter in Nitroaspirin group - Significant group difference |  |
| Harris et al., 2007 [23] | Compare FMD response to acute exercise between active and inactive overweight men | - 6 Overweight Active Men - 6 Overweight Inactive Men | - Treadmill - 45 min @ 25, 50 & 75% VO_2_peak | - Baseline - 1 hr Post | None | - Active   24% increase*   - Inactive   32% decrease* | Results for all three intensities were not different. Reported data is from the combined three intensities |
| ^*^ Significant pre-to post-exercise  ^#^ Significant difference between groups | | | | | | | |
